# Developing an AI-Based Model for Lagophthalmos and Bell's Phenomenon Detection in Intensive Care Unit Patients: A Gold Standard Comparison

**DOI:** 10.7759/cureus.101591

**Published:** 2026-01-15

**Authors:** Maram Alnefaie, Nawaf S Althobaiti, Abdulrahman Almatrafi, Haya A Alnafisah, Majed Alqurashi

**Affiliations:** 1 Ophthalmology, Alhada Armed Forces Hospital, Taif, SAU; 2 Medicine, Taif University, Taif, SAU; 3 Ophthalmology, Alnoor hospital, Makkah, Saudi Arabia, Taif, SAU; 4 Ophthalmology, Prince Mohammed bin Abdulaziz Hospital, Riyadh, SAU

**Keywords:** artificial intelligence, critically ill patients, exposure keratopathy, lagophthalmos, screening tool

## Abstract

Purpose

To validate a newly developed artificial intelligence (AI) model as a screening tool for detecting lagophthalmos and poor Bell's phenomenon, by comparing its performance with the gold-standard examination conducted by an ophthalmologist.

Design

A cross-sectional observational study was conducted. External eye photographs of patients admitted to intensive care units at a secondary hospital in Taif City, Kingdom of Saudi Arabia, were analyzed by the AI model to detect lagophthalmos and assess Bell's phenomenon.

Conclusions and importance

The findings of this study have the potential to significantly impact healthcare by enabling the prediction of serious ocular complications through an AI-based screening model for ICU patients. The integration of AI models can improve patient screening, facilitating early preventive measures such as lid tapes and lubricants to reduce the occurrence of exposure keratopathy. If validated, this AI model could be effectively utilized by non-ophthalmologist staff, including ICU nurses, thereby promoting earlier detection of lagophthalmos and preventing prolonged corneal exposure, which is a known risk factor for exposure keratopathy

## Introduction

Exposure keratopathy is a severe ocular complication caused by incomplete eye closure, leading to prolonged corneal exposure and disruption of the tear film. This condition can result in corneal abrasion, infection, scarring, and, in severe cases, permanent visual loss [1-5]. Therefore, early identification of potential etiologies is crucial for effective management.

Lagophthalmos and poor Bell's phenomenon are key clinical signs that play a significant role in predicting exposure keratopathy. Despite their importance, these conditions are often overlooked by non-ophthalmologist healthcare providers until complications, such as exposure keratopathy, arise - typically manifesting as eye redness or eyelid swelling, indicative of exposure keratopathy.

Critically ill patients-especially those who are mechanically ventilated, sedated, or have impaired blinking and lagophthalmos-are at a higher risk of developing exposure keratopathy [6-8]. Additionally, patients with a lower Glasgow Coma Scale score are more susceptible. Given these risks, an effective ophthalmologic screening tool is crucial to prevent the sequelae of exposure keratopathy.

Artificial intelligence (AI) has made significant advancements in ophthalmology, particularly in screening. AI algorithms, especially deep learning models, have demonstrated high accuracy in detecting ophthalmic conditions such as diabetic retinopathy (DR), age-related macular degeneration (AMD), and glaucoma [9-11]. These successes underscore AI's potential to enhance early diagnosis and improve patient outcomes by providing accessible, high-accuracy screening tools.

To the best of our knowledge, this is the first study to assess both lagophthalmos and poor Bell's phenomenon while performing a gold-standard comparison between an AI-based detection tool and ophthalmologists in evaluating critically ill patients.

## Materials and methods

Method

We conducted a cross-sectional observational study from June to September 2024, including 68 eyes from 34 patients admitted to the intensive care units of a secondary hospital in Taif, Saudi Arabia.

Aim

This study aimed to compare the diagnostic capabilities of ophthalmologists, non-ophthalmologists, and a newly developed artificial intelligence model in detecting lagophthalmos and assessing Bell's phenomenon, both critical clinical signs.

Artificial intelligence model training

To train the AI model, we explored multiple approaches for lagophthalmos detection, including an initial face detection step, followed by specific eye detection, and the subsequent application of a specific classification algorithm.

Our AI model employs a deep learning classification algorithm that processes images of human eyes and classifies them as either "lagophthalmos" or "normal". To uphold patient privacy, the model was exclusively trained using close-up eye images rather than full-face images.

We utilized the EfficientNet pre-trained deep learning architecture for classification, fine-tuning it on our two-class dataset. EfficientNet is a convolutional neural network (CNN) that optimally scales network depth, width, and resolution using a compound coefficient. Unlike conventional methods that scale these factors arbitrarily, EfficientNet uniformly adjusts network dimensions using predefined scaling coefficients.

**Figure 1:**
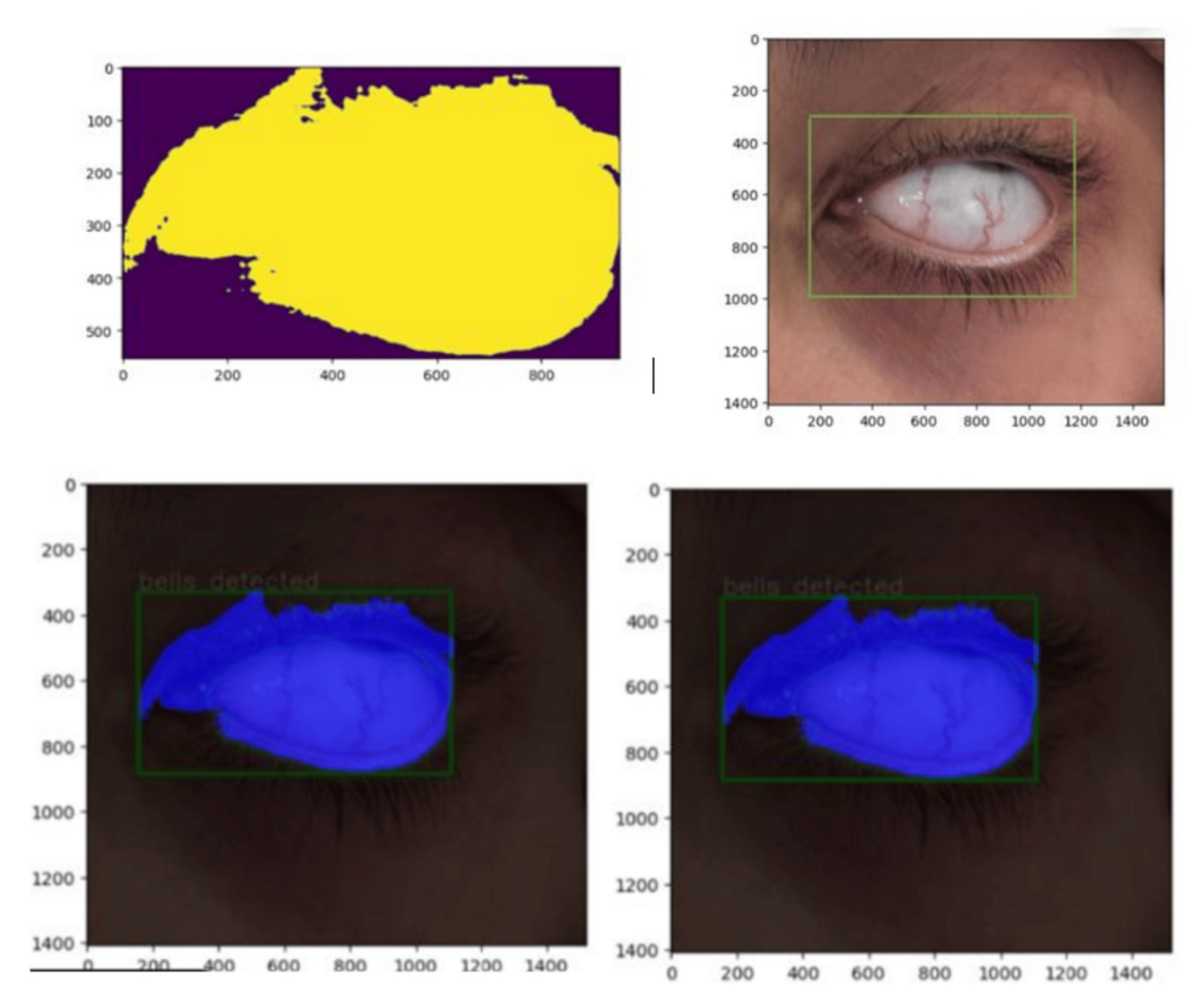
AI model training stages for eye localization, segmentation, and Bell's palsy indicators

For Bell's phenomenon training, a dedicated phase was conducted to enhance the AI model's capability in detecting this specific ocular reflex. This involved compiling a comprehensive dataset of images showcasing upward eye rotations captured under both voluntary and reflective conditions. The model was exposed to a wide range of presentations, including normal and abnormal variations of Bell's phenomenon, to ensure comprehensive learning. Expert ophthalmologists meticulously annotated these images, categorizing them based on the presence or absence of Bell's response. This meticulous approach aimed to refine the AI model's predictive accuracy and improve its clinical applicability in ICU patient screening for this critical sign (Figure 1).

Ophthalmological examination

A comprehensive ophthalmological examination was conducted by a qualified ophthalmologist for each participant. Recorded general parameters included age, gender, reason for admission, and the use of invasive ventilation, sedatives, and muscle relaxants. Ocular assessments specifically included laterality, the presence of lagophthalmos, and the quality of Bell's phenomenon.

Anterior segment examination was performed using a 20-Diopter lens and an indirect ophthalmoscope with cobalt blue light, combined with fluorescein eye drops. This assessment evaluated for conjunctival injection, superficial punctate keratitis, corneal epithelial defects, microbial keratitis, corneal perforation, band keratopathy, and corneal scarring. Patients were diagnosed with exposure keratopathy if any of the following were present: superficial punctate keratitis (SPKs), corneal epithelial defects (CED), microbial keratitis, corneal perforation, band keratopathy, or corneal scarring.

**Figure 2:**
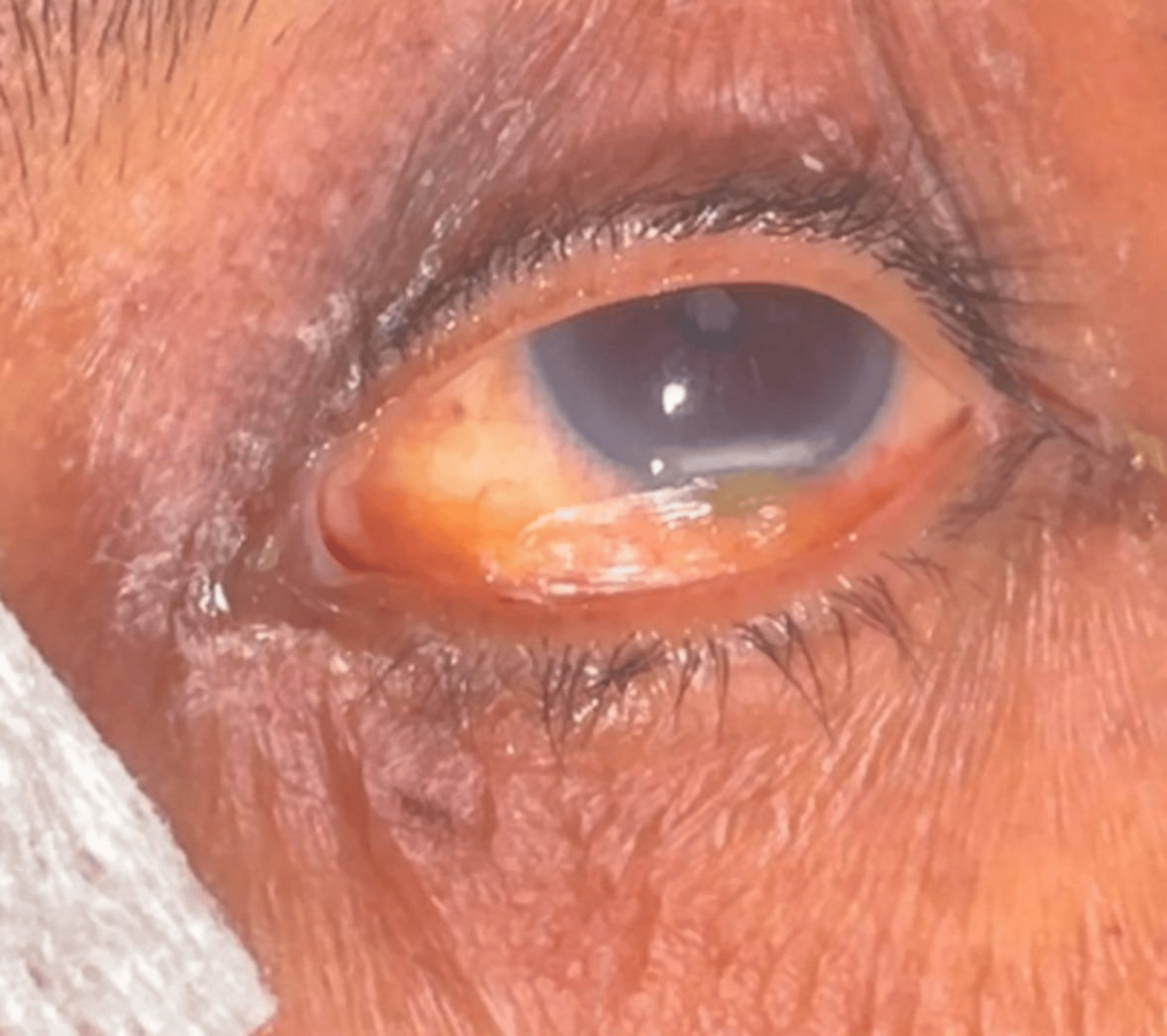
Microbial keratitis in the inferior cornea with an overlaying corneal epithelial defect and marked conjunctival injection

**Figure 3:**
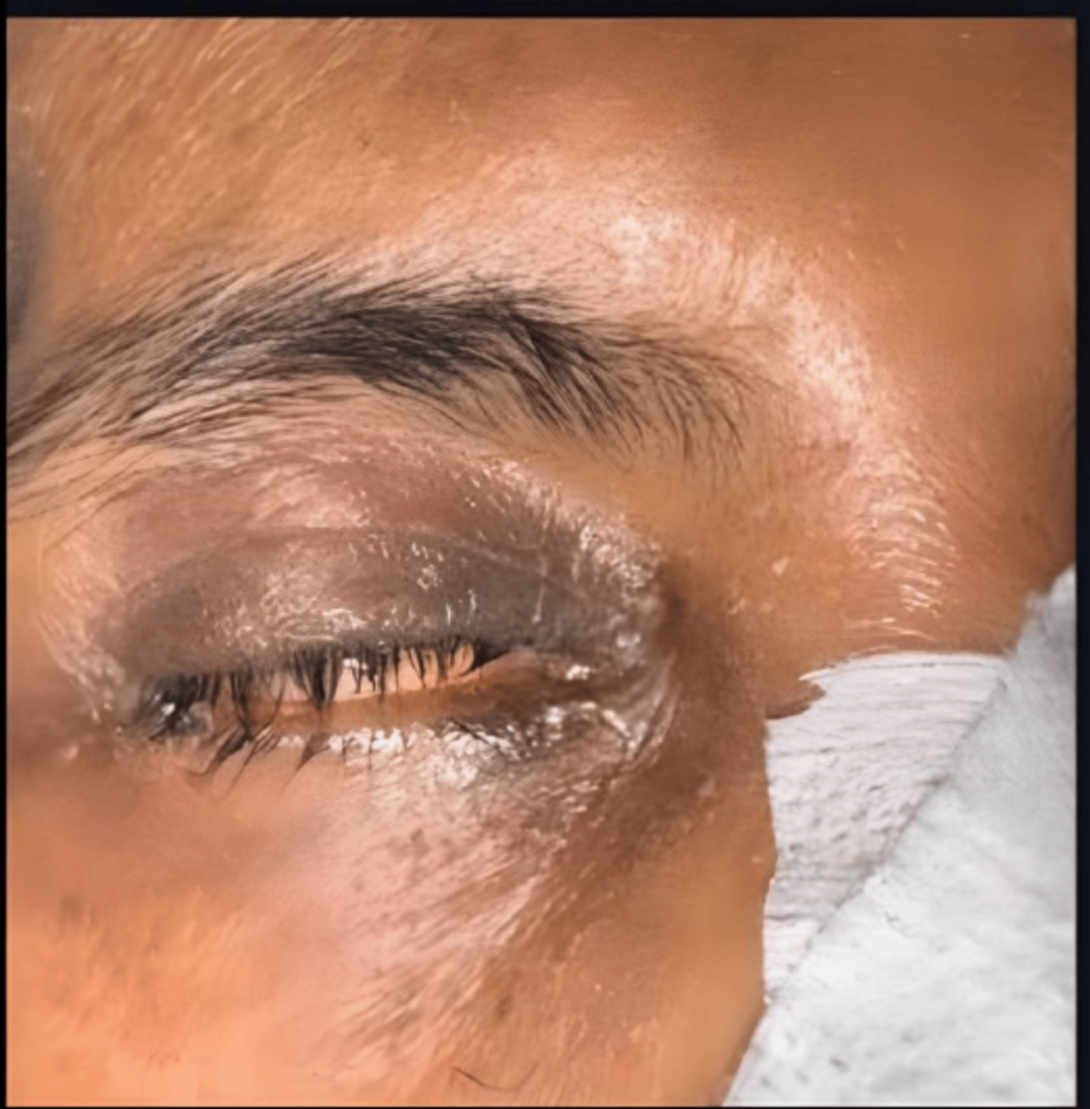
Lagophthalmos detection for the same patient by our artificial intelligence model

Each patient's eye was photographed using a single camera in JPG format for subsequent AI model analysis. Additionally, non-ophthalmologist ICU staff assessed lagophthalmos and poor Bell's phenomenon to evaluate the feasibility of non-specialists for initial screening, allowing for a comparative analysis with the AI model and ophthalmologist assessment (Figures 2, 3).

## Results

**Figure 4:**
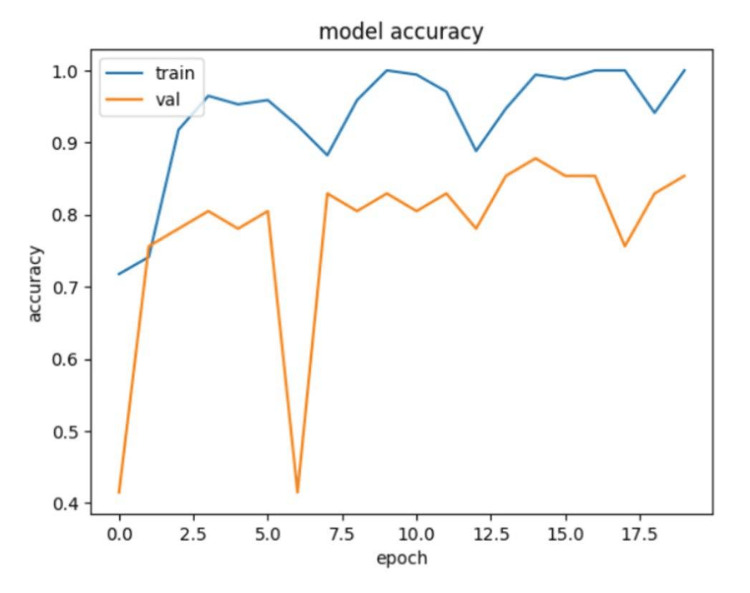
Training and validation accuracy curves for the AI model in lagophthalmos detection

We fine-tuned the model using a training dataset comprising 150 lagophthalmos samples and 50 normal samples. A graph illustrating the training process demonstrates a consistent accuracy increase in both training and validation datasets (Figure 4).

The study analyzed 68 eyes from 34 participants, with a mean age of 47 years. The sample included 18 males (54.3%) and 16 females (45.7%). Notably, exposure keratopathy was more prevalent in males, with a male-to-female ratio of 3:1. Hospital admission reasons varied, with neurological and cardiac disorders being the most common (eight participants each, 23.5%). Pulmonary diseases accounted for five admissions (14.7%), followed by endocrine disorders and traumatic brain injuries (four admissions each, 11.8%). Infectious diseases and muscular disorders were each reported in two participants (5.9%), while one participant (2.9%) had a hematological disorder. Exposure keratopathy was most prevalent in patients with muscular disorders (100%) and traumatic brain injuries (75%), as shown in Table 1. 

**Table 1:** Patients' characteristics in relation to exposure to keratopathy § P-value has been calculated using Fisher's Exact test. ‡ P-value has been calculated using an independent sample t-test. ** Significant at p<0.05 level

Study variables	Overall N (%) (n=34)	Exposure to keratopathy N (%)		p-value
		No (n=17)	Yes (n=17 )	
Age in years (mean ± SD)	47.5 ± 30.0	46.0 ± 30.4	48.9 ± 30.5	0.780 ^‡^
≤50 years	16 (47.1%)	8 (47.1%)	8 (47.1%)	1.000^§^
>50 years	18 (52.9%)	9 (52.9%)	9 (52.9%)	
Gender				0.039 **
Male	18 (52.9%)	6 (35.3%)	12 (70.6%)	
Female	16 (47.1%)	11 (64.7%)	5 (29.4%)	
Reason for admission				0.077^§^
Cardiac disorders	8 (23.5%)	2 (11.8%)	6 (35.3%)	
Neurological disorders	8 (23.5%)	6 (35.3%)	2 (11.8%)	
Pulmonary disease	5 (14.7%)	4 (23.5%)	1 (5.9%)	
Endocrine disorders	4 (11.8%)	1 (5.9%)	3 (17.9%)	
Traumatic brain injury	4 (11.8%)	1 (5.9%)	3 (17.9%)	
Infectious disease	2 (5.9%)	2 (11.8%)	0	
Muscular disorders	2 (05.9%)	0	2 (11.8%)	
Haematological disease	1 (2.9%)	1 (5.9%)	0	
Invasive ventilation				1.000^§^
No	16 (47.1%)	8 (47.1%)	8 (47.1%)	
Yes	18 (52.9%)	9 (52.9%)	9 (52.9%)	
Muscle relaxant				0.485^§^
No	32 (94.1%)	17 (100%)	15 (88.2%)	
Yes	2 (5.9%)	0	2 (11.8%)	
Sedative use				1.000^§^
No	31 (91.2%)	16 (94.1%)	15 (88.2%)	
Yes	3 (8.8%)	1 (5.9%)	2 (11.8%)	
GCS				0.787^§^
Mild	16 (47.1%)	9 (52.9%)	7 (41.2%)	
Moderate	3 (8.8%)	1 (5.9%)	2 (11.8%)	
Severe	15 (44.1%)	7 (41.2%)	8 (47.1%)	
Duration of admission in days (Mean ± SD)	304.2 ± 470.8	322.0 ± 527	286.4 ± 422	0.829 ^‡^

Among the participants, 30 (86.6%) were not mechanically ventilated, while four (13.3%) required ventilation. Muscle relaxant use was minimal, with 32 participants (94.1%) not receiving them and only two (5.9%) requiring administration. Similarly, sedative use was low, with 31 participants (91.2%) not requiring sedatives and three (8.8%) receiving them. The mean duration of hospital admission was 10.1 days, with a standard deviation of 5.26 days. Neither invasive ventilation, muscle relaxant use, nor sedative use was found to be statistically significant in the development of exposure keratopathy in our study.

Exposure keratopathy was diagnosed in 31 of 68 patients (45.6%), with superficial punctate keratitis (SPKs) being the most common finding. Corneal epithelial defects (CED) were identified in four patients (5.9%), while one patient (1.5%) developed microbial keratitis (MK). Two patients with exposure keratopathy were managed with lubricant eye drops and gel (Artelac Nighttime Gel), but treatment was insufficient in preventing disease progression (p<0.05).

Clinically detected lagophthalmos was significantly associated with exposure keratopathy (p=0.011), as shown in Table 2. Other contributing factors included poor Bell's phenomenon (p=0.001) and conjunctival injection (p<0.001), as shown in Table 3. 

**Table 2:** Ocular characteristics in relation to exposure to keratopathy § P-value has been calculated using Fisher's Exact test. ** Significant at p<0.05 level OD - Oculus Dexter (right eye); OS - Oculus Sinister (left eye); SPKS - superficial punctate keratitis; CED - corneal epithelial defects; MK - microbial keratitis

Variables	Overall N (%) (n=68)	Exposure to keratopathy N (%)		p-value
		Yes (n=33 )	No (n=35)	
Laterality				0.808^§^
OD	34 (50.0%)	17 (51.5%)	17 (48.6%)	
OS	34 (50.0%)	16 (48.5%)	18 (51.4%)	
Lagophthalmos - ophtha				0.011 **^§^
No	51 (75.0%)	20 (60.6%)	31 (88.6%)	
Yes	17 (25.0%)	13 (39.4%)	4 (11.4%)	
Bell's phenomena- ophtha				0.001 **^§^
Poor	31 (45.6%)	22 (66.7%)	9 (25.7%)	
Good	37 (54.4%)	11 (33.3%)	26 (74.3%)	
Conj injection				<0.001 **^§^
No	57 (83.8%)	22 (66.7%)	35 (100%)	
Yes	11 (16.2%)	11 (33.3%)	0	
SPKS				<0.001 **^§^
No	37 (54.4%)	2 (6.1%)	35 (100%)	
Yes	31 (45.6%)	31 (93.9%)	0	
CED				0.034 **^§^
No	64 (94.1%)	29 (87.9%)	35 (100%)	
Yes	4 (5.9%)	4 (12.1%)	0	
MK				0.485^§^
No	67 (98.5%)	32 (97.0%)	35 (100%)	
Yes	1 (1.5%)	1 (3.0%)	0	
Corneal scar				0.349^§^
No	64 (94.1%)	30 (90.9%)	34 (97.1%)	
Yes	4 (5.9%)	3 (9.1%)	1 (2.9%)	
Band keratopathy				0.349^§^
No	64 (94.1%)	30 (90.9%)	34 (97.1%)	
Yes	4 (5.9%)	3 (9.1%)	1 (2.9%)	
Medical management				0.006 **^§^
No	58 (85.3%)	24 (72.7%)	34 (97.1%)	
Yes	10 (14.7%)	9 (27.3%)	1 (2.9%)	
Eyelid taping				1.000^§^
No	66 (97.1%)	32 (97.0%)	34 (97.1%)	
Yes	2 (2.9%)	1 (3.0%)	1 (2.9%)	
Non-ophtha lagophthalmos				0.051^§^
No	61 (89.7%)	27 (81.8%)	34 (97.1%)	
Yes	7 (10.3%)	6 (18.2%)	1 (2.9%)	
Non-ophtha poor Bell's				0.478^§^
No	59 (86.8%)	30 (90.9%)	29 (82.9%)	
Yes	9 (13.2%)	3 (9.1%)	6 (17.1%)	
AI- Lagophthalmos				0.431^§^
No	20 (29.4%)	08 (24.2%)	12 (34.3%)	
Yes	48 (70.6%)	25 (75.8%)	23 (65.7%)	

**Table 3:** Logistics regression analysis for predicting EK in lagophthalmos - ophtha and poor Bell's (n=68) ** Significant at p<0.05 level EK - exposure keratopathy

Factor	OR	95% CI	p-value
Presence of lagophthalmos - ophtha			0.011 **
No	Ref		
Yes	5.037	1.438 – 17.648	
Presence of poor Bell's ophtha			0.001 **
No	Ref		
Yes	5.778	2.026 – 16.479	
Presence of both lagophthalmos and poor Bell's ophtha			0.092
No	Ref		
Yes	3.413	0.820 – 14.211	

The AI tool demonstrated a sensitivity of 94.2% in detecting lagophthalmos, significantly outperforming non-ophthalmologist examination (sensitivity: 29.4%). However, the AI model had lower specificity (62.7%). Poor Bell's-non-ophtha has a zero sensitivity (specificity: 82.4%), while lagopthalmos-AI has a sensitivity of 94.1% (specificity: 37.3%) toward the presence of lagophthalmos-ophtha, as shown in Table 4. 

**Table 4:** Sensitivity and specificity of lagophthalmos ophtha Sensitivity and specificity were calculated for the diagnostic value of lagophthalmos toward the presence of lagophthalmos-ophtha

Lagophthalmos - AI	Lagophthalmos - optha			
	True Positive 16	False Positive 32	Sensitivity (%)	Specificity(%)
	False negative 1	True negative 19	94.1%	37.3%

For the Bell's phenomenon detection tool, while some images initially posed technical challenges regarding eye segmentation, the AI model demonstrated promising value and showed good results in a significant subset of the analyzed patients where segmentation was successfully implemented, exhibiting high sensitivity.

## Discussion

Exposure keratopathy (EK) is a common ocular surface disorder in critically ill patients, resulting from tear film instability and incomplete eyelid closure. It can lead to varying degrees of corneal damage, increasing the risk of complications. Studies suggest that EK affects both males and females equally. The overall reported incidence of EK is approximately 21%, though it is significantly higher among mechanically ventilated patients (up to 54.3%). In contrast, EK incidence in patients receiving non-invasive ventilation or without ventilatory support is notably lower (5.1%). Other studies have reported incidence rates as high as 40%, while a 2008 meta-analysis estimated a range of 20% to 42%.

Critically ill patients have an increased risk of EK due to both intrinsic and iatrogenic factors. Patient-related factors include decreased consciousness, reduced tear production, lower blink rate, impaired corneal reflex, incomplete eye closure, and increased vascular permeability. Iatrogenic contributors include mechanical ventilation, sedation, and muscle relaxant use [12-14].

Our study found a high prevalence of exposure keratopathy, particularly superficial punctate keratitis, in patients without lagophthalmos, accounting for up to 50% of the total study population. This finding contrasts with a study by Araujo et al. (2016) conducted at a public teaching hospital in Brazil. [8] The increased prevalence in our study may be attributed to severe ocular surface dryness, which can be the result of several factors in the absence of lagophthalmos, such as a significant reduction in lacrimation, poor blinking, and other confounding variables.

Artificial intelligence (AI) is increasingly utilized in ophthalmology for the automated detection of vision-threatening diseases. The FDA-approved AI system IDx-DR, for instance, can autonomously identify diabetic retinopathy in retinal images, demonstrating a sensitivity of 87.2% and specificity of 90.7% . Additionally, AI models have shown comparable or superior performance to human experts in detecting age-related macular degeneration (AMD) from retinal fundus images, with one study reporting an accuracy exceeding 92%. In glaucoma screening, AI models using optic nerve head imaging have achieved sensitivities ranging from 83% to 94% for detecting glaucomatous changes.

Our AI model exhibited high sensitivity in detecting lagophthalmos but had relatively lower specificity. The reduced specificity may be attributed to the limited number of training images depicting normal eyelid closure, suboptimal image lighting, and the presence of skin folds in lower eyelids.

For the Bell's tool, not all images could be analyzed due to technical issues in eye segmentation. However, in the images where segmentation was successfully implemented without error, the tool demonstrated high sensitivity.

A limitation of this study was the restricted training dataset, particularly regarding images of normal eyelid closure. This highlights the need for a more extensive dataset to improve specificity and enhance the model's performance

## Conclusions

The results of this study highlight the significant impact of lagophthalmos on the occurrence of exposure keratopathy, underscoring the importance of early detection and intervention. Timely initiation of preventive measures, such as lubricants or eyelid closure techniques, can effectively reduce the risk of keratopathy. Therefore, the role of accurate and accessible screening becomes even more critical.

Our AI-based screening tool provides a valuable contribution by enabling non-ophthalmologists, such as ICU nurses and general practitioners, to easily identify at-risk patients. By facilitating earlier detection, this tool has the potential to improve patient outcomes and reduce the incidence of exposure keratopathy in critically ill patients. 
